# Community ecology across bacteria, archaea and microbial eukaryotes in the sediment and seawater of coastal Puerto Nuevo, Baja California

**DOI:** 10.1371/journal.pone.0212355

**Published:** 2019-02-14

**Authors:** Sabah Ul-Hasan, Robert M. Bowers, Andrea Figueroa-Montiel, Alexei F. Licea-Navarro, J. Michael Beman, Tanja Woyke, Clarissa J. Nobile

**Affiliations:** 1 Department of Molecular and Cell Biology, School of Natural Sciences, University of California Merced, Merced, CA, United States of America; 2 Quantitative and Systems Biology Graduate Program, University of California Merced, Merced, CA, United States of America; 3 Department of Energy, Joint Genome Institute, Walnut Creek, CA, United States of America; 4 Department of Biomedical Innovation, Centro de Investigación Científica y de Educación Superior de Ensenada (CICESE), Ensenada, Baja California, México; 5 Department of Life and Environmental Sciences, School of Natural Sciences, University of California Merced, Merced, CA, United States of America; Free University of Bozen/Bolzano, ITALY

## Abstract

Microbial communities control numerous biogeochemical processes critical for ecosystem function and health. Most analyses of coastal microbial communities focus on the characterization of bacteria present in either sediment or seawater, with fewer studies characterizing both sediment and seawater together at a given site, and even fewer studies including information about non-bacterial microbial communities. As a result, knowledge about the ecological patterns of microbial biodiversity across domains and habitats in coastal communities is limited–despite the fact that archaea, bacteria, and microbial eukaryotes are present and known to interact in coastal habitats. To better understand microbial biodiversity patterns in coastal ecosystems, we characterized sediment and seawater microbial communities for three sites along the coastline of Puerto Nuevo, Baja California, Mexico using both 16S and 18S rRNA gene amplicon sequencing. We found that sediment hosted approximately 500-fold more operational taxonomic units (OTUs) for bacteria, archaea, and microbial eukaryotes than seawater (p < 0.001). Distinct phyla were found in sediment versus seawater samples. Of the top ten most abundant classes, Cytophagia (bacterial) and Chromadorea (eukaryal) were specific to the sediment environment, whereas Cyanobacteria and Bacteroidia (bacterial) and Chlorophyceae (eukaryal) were specific to the seawater environment. A total of 47 unique genera were observed to comprise the core taxa community across environment types and sites. No archaeal taxa were observed as part of either the abundant or core taxa. No significant differences were observed for sediment community composition across domains or between sites. For seawater, the bacterial and archaeal community composition was statistically different for the Major Outlet site (p < 0.05), the site closest to a residential area, and the eukaryal community composition was statistically different between all sites (p < 0.05). Our findings highlight the distinct patterns and spatial heterogeneity in microbial communities of a coastal region in Baja California, Mexico.

## Introduction

The identification and description of microbial biodiversity patterns is important for understanding the biological underpinnings of ecosystem function. This is particularly true for coastal microbial communities, as they play important roles in the regulation of biogeochemical cycling at the land-sea interface [1,2], and in the ecological dynamics of larger organisms through symbiosis and disease [3,4]. Coastal microbial communities are complex and spatially variable [5–7], consisting of all domains of life interacting with each other in the water column and sediment [8]. The heterogeneity of coastal microbial communities thus demands intensive sampling to improve our understanding of microbial ecology and the structure and function of coastal ecosystems. Many studies of coastal microbial communities, however, take place along waters of Western world countries or at somewhat subjective “exotic” locales [9]. This leaves large swaths of un-sampled/under-sampled coastlines around the world where microbial diversity–and its associated geochemical and physical diversity–is poorly characterized.

A surge in marine microbial community ecology research over the past decade has led to a wealth of new information on the dynamics between microorganisms and their surrounding environments [6]. As a result, the identification of spatial and temporal patterns of microbial diversity, and how this information correlates to biogeochemical cycling, has been vastly expanded [10–17]. A recent commentary by Brussaard and colleagues, for example, highlights the growing roles that “big data” from microbial ecology and biogeochemistry studies play in understanding how microbial communities shape the biogeochemical cycling patterns of coasts and oceans [18]. Such information gathered over time provides a starting point to determining the causes and effects of microbial community disturbances [19]. While these discoveries are innovative by providing new insight into marine microbial ecosystems, much coastal microbial diversity remains uncharacterized [20–22].

The majority of microbial biodiversity “omics” studies are overwhelmingly focused on bacterial communities using 16S rRNA amplicon sequencing, and are often limited to a specific environment type rather than considering multiple aspects of microbial ecosystems [23,24]. Coastal microbial communities present a dynamic assemblage to test taxa richness and diversity between two environment types: sediment solids and seawater liquids. As a result of its texture, soil is well known to host high microbial richness across domains [25] and, by extrapolation, this is also likely to be the case for sediment [26] since sediment also possesses a large surface area for microorganisms to attach [27]. The added value of using next-generation technology with these types of sampling studies is that it provides detailed information on taxa within a larger ecosystem framework.

Investigating the sediment and seawater at one coastal point using biological replicates is advantageous because it allows for the comparisons of species richness estimates and abundance profiles across sample types [28]. Taking these measurements into account, an ecological study of microbial mats, for example, observed that bacterial and archaeal mat biodiversity in intertidal, hypersaline, and hot spring environments was influenced by mat chemistry and spatial location, more so than by temporal changes [29]. These variations between locations can correspond to variations in function and/or recovery after perturbation [30], and thus emphasize the importance of simultaneously characterizing both richness and abundance measurements in microbial ecology studies.

While the Baja California coastline shares the same marine ecoregion with the United States [31], its microbial biodiversity is surprisingly understudied relative to the Southern Californian coastline [32]. The Southern California Bight ecoregion of Baja California experiences intense upwelling events that are predicted to increase with climate change [33,34], and thus undergoes substantial nutrient flux that could affect microbial composition [35]. The handful of existing microbial biodiversity next-generation sequencing studies on the Baja California coast are largely centered on the hypersaline environments throughout Guerrero Negro, which differ considerably from coastal environments in terms of community composition [36–44]. We selected the coastal site of Puerto Nuevo in Baja California, which is close to the United States-Mexico border, for the following reasons. First, this region experiences strong upwelling events that are associated with nutrient fluxes. Such upwelling events also lead to marine organism habitat loss, and are increasing with climate change [33,34]. Second, this region shares overlapping coastal physical features with Southern California and is thus likely to share similarities in microbial ecosystems. Third, this location is unrepresented in terms of coastal microbial community sampling, thus its study would expand our existing knowledge of microbial diversity. With these reasons in mind, the primary goal of our study is to obtain information on coastal microbial diversity across domains and environment types in Puerto Nuevo to set the precedent for additional microbial ecology studies along the Baja California coastline.

Using high-throughput sequencing, we characterized the bacterial, archaeal, and eukaryal microbial diversity in the sediment and seawater of three sites along a 0.45 km range in Puerto Nuevo in Playas de Rosarito, Baja California. Our goals were to determine (1) the differences in coastal microbial community richness and/or abundance between seawater and sediment environment types, (2) the alpha diversity within a sampling site versus the beta diversity among a 0.45 km range, and (3) the shared versus unique patterns between bacterial, archaeal and eukaryal microbial communities.

## Materials and methods

### Study area and sampling

The necessary field permit for this study (permit # PPF/DGOPA-009/17) was issued from the Secretaría de Agricultura, Ganadería, Desarrollo Rural, Pesca y Alimentación (SAGARPA), complying with all relevant regulations.

**Fig 1:**
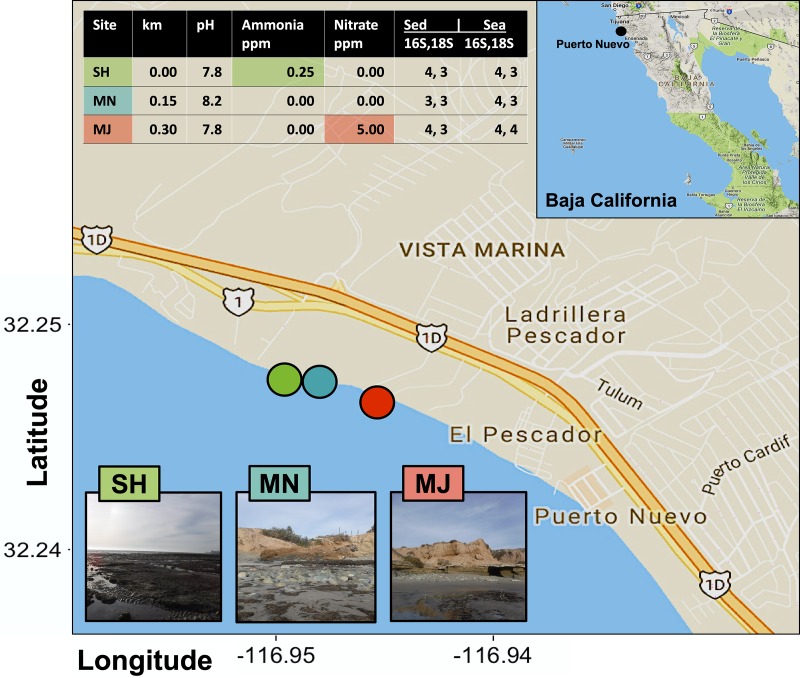
Location and metadata information of sampling sites. The three sampling sites are denoted in lime green (SH or sheltered), cyan (MN or Minor Outlet) and red (MJ or Major Outlet) circles. Sequenced samples based on seawater or sediment are displayed in the right-hand table columns for a total sequence output of 42 out of 48 samples submitted. The inset illustrates the approximate sampling location within Baja California, as denoted with a black circle. Chemical differences unique to sites are highlighted using colored boxes in the upper inset table and km refers to the distance in kilometers that MN and MJ are relative to SH.

The coastal Puerto Nuevo site is a fishing community near Playas de Rosarito that is frequently visited by tourists and covered in *Zostera* eel grass beds. We selected three sampling sites at low tide (~1 m in depth each) on the Puerto Nuevo coastline with gradient exposures to human impact along a 0.45 km range between 32.248 N, -116.948 E and 32.246 N, -116.944 E (Fig 1). We refer to the most North-facing site at point 0.0 km as the Sheltered (SH) site, the site at point 0.15 km as the Minor Outlet (MN) site, and the site at point 0.3 km as the Major Outlet (MJ) site. The SH site is facing a 5–7 m cliff at point 0.0 km, the MN site is near a small run off outlet or scour at point 0.15 km, and the MJ site is near a large run off outlet and residential area at point 0.3 km. Four replicates of surface seawater samples and sediment core samples were collected at each site according to previously described methods [45]. Salinity, temperature (°C), pH, ammonia (ppm), nitrite (ppm), and nitrate (ppm), were measured for each site using the API Saltwater Master Test Kit.

Seawater samples (200 mL) were filtered on-site using sterile 60 mL syringes with 25 mm hydrophilic polyethersulfone 0.1-micron membrane filters (Supor-200 PES; Pall Laboratories) at an approximate rate of 15 mL/min. Filters were then transferred into individual, sterile 2 mL Eppendorf tubes, immediately frozen on dry ice, and stored at -80°C until further processing. For sediment cores, the tips of sterile 8.5 cm length x 1.5 cm diameter syringes were cut using sterile razor blades prior to being vertically inserted into the sediment. Sediment samples were then kept in their respective syringes and wrapped with Parafilm, immediately frozen on dry ice, and stored at -80°C until further processing. All samples were handled with sterile nitrile gloves both on- and off-site.

### DNA extraction, PCR amplification for validation, and Illumina amplicon sequencing

DNA from the filters of 200 mL seawater samples was extracted using the QIAGEN DNeasy Blood & Tissue Kit (Qiagen, Valencia, CA, United States) following the manufacturer’s protocol. Filters were cut into 2 mm strips using sterilized scissors and the microbial content on the filter was homogenized using the Omni Bead Ruptor homogenizer (Omni International, Kennesaw, GA, United States) with a mixture of 0.1, 0.5, and 1.4 micron beads to maximize retrieval of DNA from all microbial domains. DNA from sediment samples was extracted from 0.5 g of field-moist sediment using the MoBio PowerSoil DNA Isolation Kit (MoBio, Carlsbad, CA, United States) following the manufacturer’s protocol. All extracted DNA from seawater and sediment samples was diluted to a final concentration of 5 ng per μL each.

Ribosomal RNA gene amplification was performed for all samples, including a variable 12 bp barcode sequence to ensure that samples were uniquely identifiable, following a standard protocol from the Department of Energy Joint Genome Institute (JGI) [46]. The V4-V5 region for 16S rRNA of bacteria and archaea (FW 515 F 5’- GTGYCAGCMGCCGCGGTAA-3’, RV 926R 5’- CCGYCAATTYMTTTRAGTTT-3’) and the V4 region for the 18S rRNA of eukaryotes (FW 5’- CCAGCASCYGCGGTAATTCC-3’, RV 5’- ACTTTCGTTCTTGATYRA-3’) were targeted, with sample validation amplifications to assess extraction quality [47–51]. Stocks of 2x AccuStart II PCR SuperMix containing Taq DNA Polymerase (Quantabio, Beverly, MA, United States) and 10 mg/mL bovine serum albumin (BSA) (ThermoFisher Scientific, Waltham, MA, United States) were used during PCR amplification validation checks, conducted prior to amplicon sequencing. A final concentration of 1x SuperMix and 10 μg BSA was used for each 25 μL PCR reaction containing 10 ng DNA, 500 nM each for a given forward and reverse primer (1 μM total), and the remaining PCR reaction volume was made up to 25 μL with PCR grade nuclease-free water. The 16S rRNA region was amplified by denaturation at 94°C/3 min, followed by 30 cycles of denaturation 94°C/30 sec, annealing at 50°C/30 sec, elongation at 72°C/1 min, and a final elongation 72°C/10 min. The 18S rRNA region was amplified by denaturation at 94°C/3 min, followed by 30 cycles of denaturation 94°C/30 sec, annealing at 60°C/30 sec, elongation at 72°C/1.5 min, and a final elongation 72°C/10 min. After validation, 250 ng of extracted DNA in 50 μL total volume was used for plate-based next-generation 16S and 18S amplicon sequencing at the JGI using a KAPA Biosystem library qPCR kit and a Roche LightCycler 480 real-time PCR instrument with the same primers; a MiSeq Reagent kit using a 2x300 nt indexed protocol was used for sequencing on the Illumina MiSeq platform (Illumina, San Diego, CA, United States) [52]. Additional details for similar 16S and 18S sequencing protocols can be found on protocols.io: dx.doi.org/10.17504/protocols.io.nuudeww [53] and dx.doi.org/10.17504/protocols.io.nuvdew6, respectively [54].

### Sequence processing

Raw sequences were de-multiplexed and clustered into Operational Taxonomic Units (OTUs) using the iTagger v1.2 [49] and QIIME2 [55] pipelines for quality control and sequence analyses. Taxonomy was assigned by 97% identity or higher via the Silva database SSU for the 16S marker and SSU for the 18S r108 marker [46,49]. Identified and matched sequences were additionally filtered to remove mitochondrial DNA sequences. All remaining 16S and 18S rRNA gene sequences, with the sample having the lowest number of reads being 141944, were then rarefied at 1,000 reads per sample (23 output x 1,000 = 23,000 for 16S rRNA total rarefied reads and 21 output x 1,000 = 21,000 for 18S rRNA total rarefied reads; Fig 1 and S1 Fig). In sum, we submitted 24 samples for 16S and 18S sequencing (12 for seawater and 12 for sediment, containing 4 biological replicates per site), with an output of 23 datasets for 16S and 21 datasets for 18S.

### Data analyses and statistics

Singleton and doubleton reads were removed before creating the two datasets per rRNA region (four in total). The four datasets include read abundance or presence-absence data, with 16S and 18S for each. The first dataset created was read abundance and the second was a conservative “presence = 1” or “absence = 0” assignment of rarefied reads (GitHub Supplemental-Results.Rmd code available at https://github.com/sabahzero/Puerto-Nuevo_Coastal-Microbial-Ecology_16S-18S-Workflow_UlHasan-etal). These metrics were then used to determine the biodiversity of each site (alpha diversity) and among sites (beta diversity). Abundant phyla and classes were classified and ranked into respective taxonomic groups. For diversity, we utilized Shannon’s and Simpson’s diversity indices based on read abundance. For abundance, we compared rarefied OTU reads of taxa by log fold. For richness, we assigned taxa as present or absent, then compiled taxa by phylogenetic group (i.e. phyla, class, order). For core taxa as indicators of the community, we took a DESeq2-like approach and compared taxa richness 16S or 18S across all samples versus sediment and seawater environment types versus SH, MN, and MJ site locations in order to define core taxa between three total categories: Puerto Nuevo core taxa, core taxa of environment type, and core taxa of location.

All statistical tests and visualizations were conducted in R [56] with all code and package citation information available at https://github.com/sabahzero/Puerto-Nuevo_Coastal-Microbial-Ecology_16S-18S-Workflow_UlHasan-etal (S1–S8 Tables). Changes in microbial community structure among sites were analyzed using permutational multivariate analysis of variance (PERMANOVA; [57]) with Bray-Curtis distances [58] for the abundance datasets and Jaccard indices [59] for the richness datasets. A Bonferroni p-value correction was used to determine pairwise differences between sites. Beta diversity differences in community structure and associated statistics were visualized using Venn diagrams and proportion of variance for principal components analysis (PCA) along two axes, grouped by environment type (sediment or seawater) versus location. For all univariate data, we used analysis of variance (ANOVA) to determine significant differences among sites, environment type, and site*environment type interactions. We used q-q plots and scale-location plots to inspect normality and homoscedasticity, respectively. Where significant differences were detected, Tukey’s Test of Honest Significant Differences was used to determine the range of differences among the sites and interactions.

## Results

### Coastal Puerto Nuevo sample site metadata

We sampled four replicates of sediment and seawater from three sites within a 0.45 km range off the Puerto Nuevo coastline (Fig 1) and collected associated metadata at the interface where seawater meets sediment. The pH (7.9 ± 0.2), ammonia (0.08 ± 0.14 ppm) and nitrate (1.7 ± 2.9 ppm) levels, as well as temperature (15.8 ± 0.3°C) varied between sites during sampling in June 2016, whereas salinity (1.02 ± 0.00 psu), nitrite (0.0 ± 0.0 ppm), and depth (1.0 ± 0.0 m) were constant (S6 Table).

### Microbial community diversity richness and abundance

A total of 14,137,026 raw reads were recovered from 23 of the 24 submitted seawater and sediment samples with median lengths of ~380 bp, publicly accessible upon free registration at the Joint Genome Institute Genome Portal, ID 502935 (S5 Table). 16S rRNA gene sequences were recovered for 11 of the 12 sediment samples (1,960,774 reads) and all of the 12 seawater samples (2,156,286 reads) for a total of 4,117,060 raw reads. 18S rRNA sequences were recovered for 9 of the 12 sediment samples (3,682,950 reads) and all of the 12 seawater samples (6,337,016 reads) for a total of 10,019,966 raw reads.

**Fig 2:**
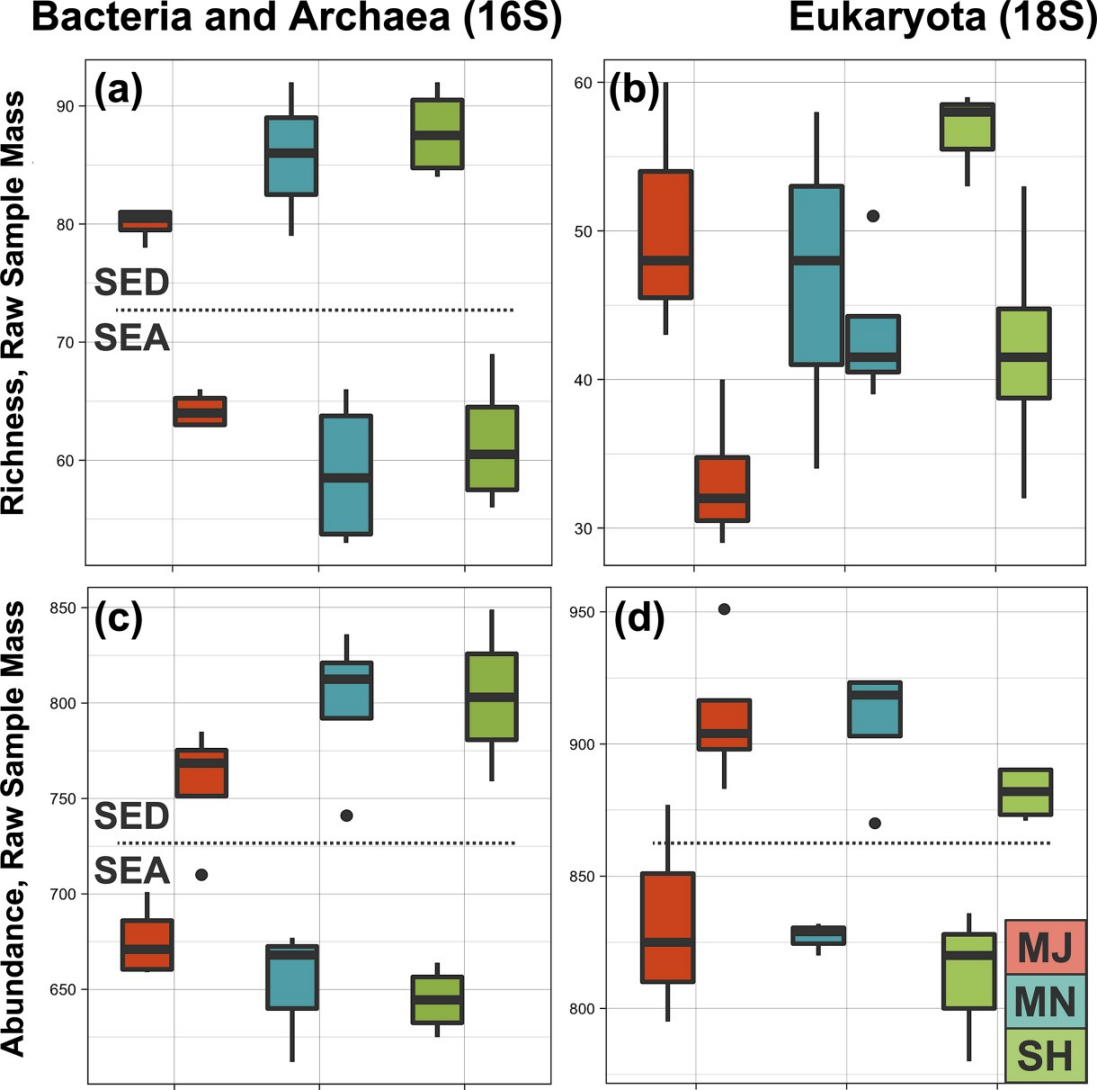
Microbial community richness and abundance. (a-b) Boxplot comparisons of rarefied bacterial and archaeal (16S) and eukaryal (18S) richness estimates for different environment types (sediment and seawater) and by sampling site (major outlet/MJ, minor outlet/MN, sheltered/SH), with a p value (p < 0.001) for environment type. (c-d) Boxplot comparisons of rarefied bacterial and archaeal (16S) and eukaryal (18S) abundance estimates for different environment types (sediment and seawater) and by sampling site (major outlet/MJ, minor outlet/MN, sheltered/SH), with a p value (p < 0.001) for environment type.

Shannon and Simpson diversity indices were produced from rarefied reads (S1 Fig), and all rarefied datasets passed the Shapiro-Wilk normality test (see GitHub Supplemental-Results.Rmd code available at https://github.com/sabahzero/Puerto-Nuevo_Coastal-Microbial-Ecology_16S-18S-Workflow_UlHasan-etal). The environment type (sediment or seawater) was found to be statistically significant for all 16S and 18S richness and abundance datasets (p < 0.005). Location was not statistically significant for any of the datasets, meaning that SH, MN or MJ did not significantly vary, although there was a correlation between environment type and site location observed for the 16S abundance dataset (p = 0.06). Focusing on environment type (sediment or seawater), analyses of reads by taking into account either raw or normalized sample mass indicated that microbial communities for sediment were orders of magnitude richer (approximately 500-fold) relative to those of seawater (Fig 2), regardless of how the data were analyzed. Taxa across domains are 2 fold richer and abundant in the sediment compared to seawater environment type. The sediment had 5.0x10^2 fold greater bacterial and archaeal taxa richness and 3.9x10^2 greater eukaryal taxa richness relative to seawater after normalization by mass. The sediment had 3.0x10^2 fold greater bacterial and archaeal taxa abundance and 2.7x10^2 greater eukaryal taxa abundance relative to seawater after normalization by mass.

### Microbial community composition

**Fig 3:**
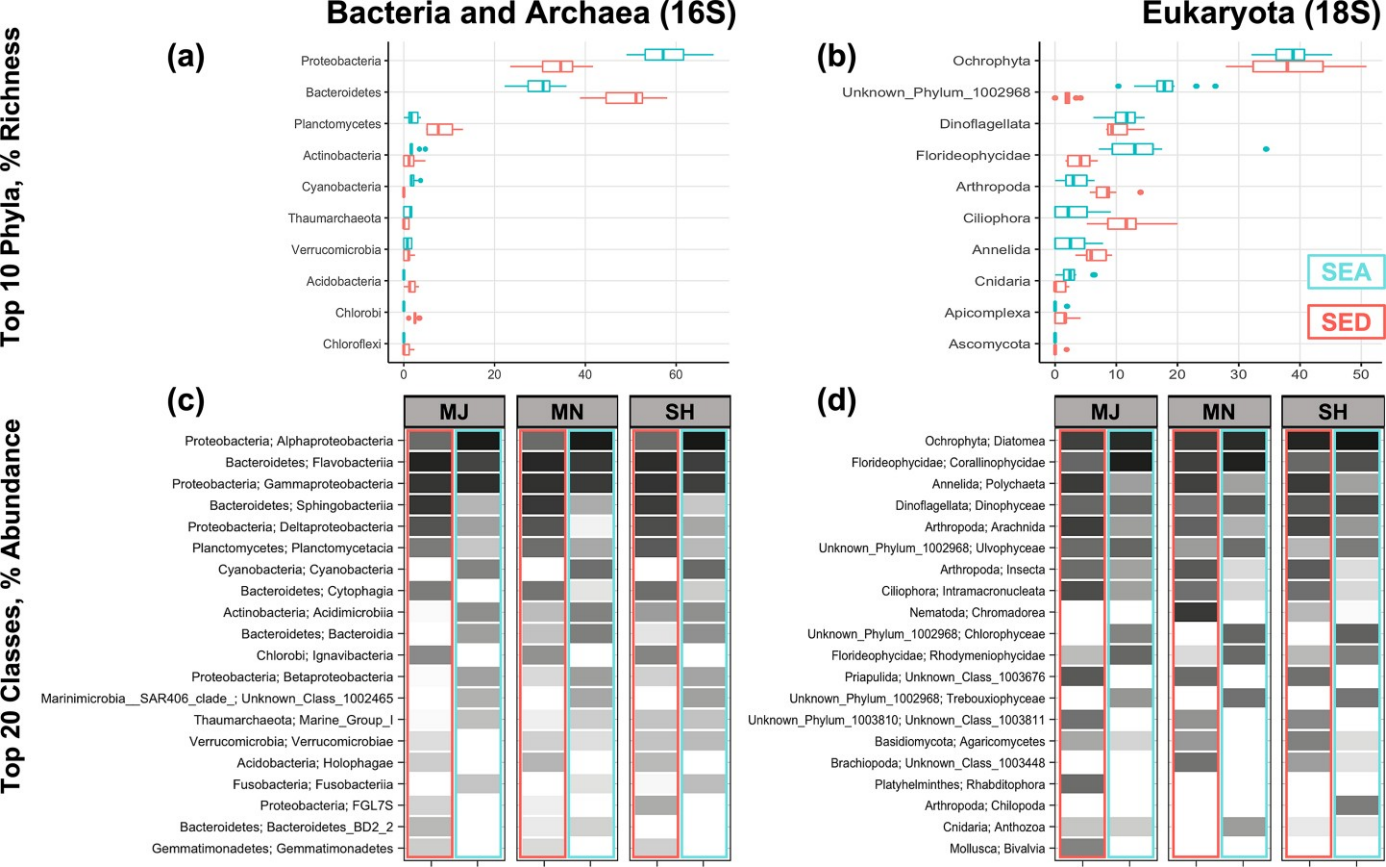
Microbial phyla by richness and abundance. Top phyla by richness differ by environment type, with some abundance specificity by site location. (a-b) The top 10 bacterial and archaeal as well as eukaryal phyla across biological replicates of site locations for both seawater (SEA) and sediment (SED) environment types from most to least richness in the sediment in order of highest relative abundance to lowest. (c-d) The top 20 bacterial and archaeal as well as eukaryal classes, demonstrating variation in abundance by environment type and site location from a gray-scale gradient of white (0) to black (100). Common or rare phyla and classes can be additionally viewed by comparison of OTU tables.

**Fig 4:**
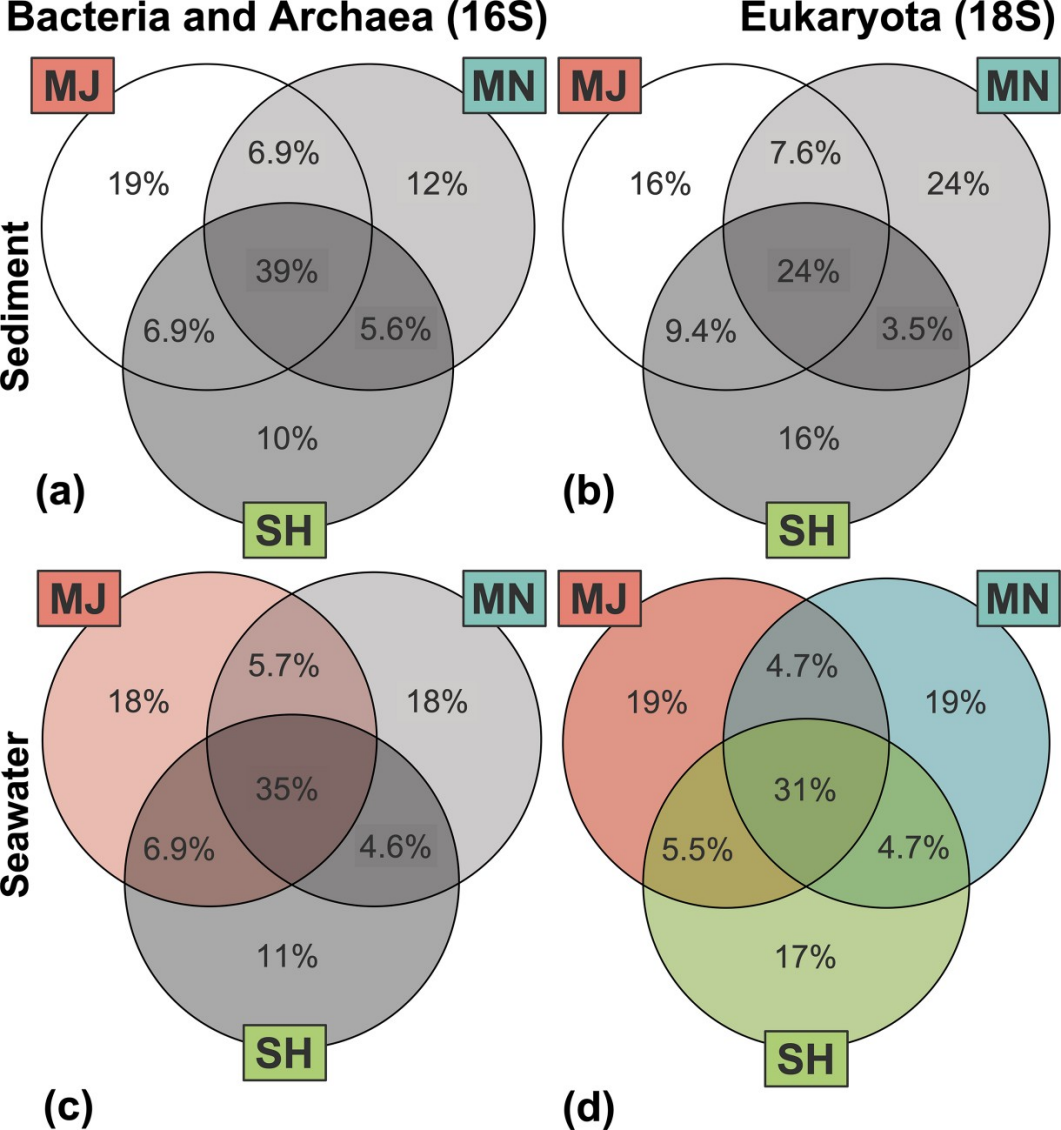
Microbial community composition and beta-diversity. Venn diagrams of percentage overlapping OTUs for bacterial and archaeal as well as eukaryal microbial communities based on richness, with site location colors corresponding to Fig 1. Statistically significant variation by PERMANOVA and pairwise comparison tests is shown in color whereas similarities (no variation) are shown as gray-scale.

In total, the Puerto Nuevo microbial community composition during the time of sampling was comprised of 3 domains: Archaea, Bacteria, and Eukarya. For prokaryotes, there were 50 phyla, 130 classes, 240 orders, 441 families, and 859 genera represented. For eukaryotes, there were 30 phyla, 56 classes, 130 orders, 165 families, and 317 genera represented. Microbial communities revealed specific taxonomic assemblages associated with sediment versus seawater samples collected from the same sites (Figs 3 and 4, Table 1). Similar to OTU richness and abundance, PERMANOVA statistics indicated that microbial community composition differed by environment type (p = 0.001, f = 68.06 for prokaryote 16S and f = 25.09 for eukaryote 18S).

**Table 1:** Shared (core) and unique core taxa for environment type and location.

Domain	Phylum	Class	Order	Family	Genus		Core
Eukaryota	Florideophycidae	Corallinophycidae	Unknown 1003045	Unknown 1003046	Mesophyllum		**ALL**
Eukaryota	Ochrophyta	Diatomea	Coscinodiscophytina	Fragilariales	Licmophora		**ALL**
Eukaryota	Ochrophyta	Diatomea	Bacillariophytina	Bacillariophyceae	Psammodictyon		**ALL**
Bacteria	Bacteroidetes	Flavobacteriia	Flavobacteriales	Flavobacteriaceae	uncultured 1216		**ALL**
Bacteria	Actinobacteria	Acidimicrobiia	Acidimicrobiales	OM1 clade	Candidatus Actinomarina		**SEA**
Bacteria	Proteobacteria	Alphaproteobacteria	Rhodobacterales	Rhodobacteraceae	Amylibacter		**SEA**
Bacteria	Proteobacteria	Alphaproteobacteria	SAR11 clade	Surface 1	Candidatus Pelagibacter		**SEA**
Bacteria	Proteobacteria	Alphaproteobacteria	Rhodobacterales	Rhodobacteraceae	Lentibacter		**SEA**
Bacteria	Proteobacteria	Alphaproteobacteria	Rhodobacterales	Rhodobacteraceae	Sulfitobacter		**SEA**
Bacteria	Proteobacteria	Alphaproteobacteria	Rickettsiales	SAR116 clade	Unknown 1000731		**SEA**
Bacteria	Proteobacteria	Alphaproteobacteria	SAR11 clade	Surface 2	Unknown 1000744		**SEA**
Bacteria	Proteobacteria	Betaproteobacteria	Methylophilales	Methylophilaceae	OM43 clade		**SEA**
Bacteria	Cyanobacteria	Cyanobacteria	SubsectionI	FamilyI	Synechococcus		**SEA**
Eukaryota	Ochrophyta	Diatomea	Bacillariophytina	Bacillariophyceae	Frustulia		**SEA**
Eukaryota	Dinoflagellata	Dinophyceae	Gymnodiniphycidae	Gymnodinium clade	Spiniferodinium		**SEA**
Bacteria	Bacteroidetes	Flavobacteriia	Flavobacteriales	Flavobacteriaceae	NS4 marine group		**SEA**
Bacteria	Bacteroidetes	Flavobacteriia	Flavobacteriales	Flavobacteriaceae	NS5 marine group		**SEA**
Bacteria	Bacteroidetes	Flavobacteriia	Flavobacteriales	Flavobacteriaceae	Polaribacter		**SEA**
Bacteria	Proteobacteria	Gammaproteobacteria	Oceanospirillales	JL ETNP Y6	Unknown 1000942		**SEA**
Bacteria	Proteobacteria	Gammaproteobacteria	Oceanospirillales	SAR86 clade	Unknown 1000950		**SEA**
Eukaryota	Unknown 1002968	Trebouxiophyceae	Chlorellales	Unknown 1003011	Nannochloris		**SEA**
Bacteria	Proteobacteria	Alphaproteobacteria	Caulobacterales	Hyphomonadaceae	Robiginitomaculum		**SED**
Bacteria	Proteobacteria	Alphaproteobacteria	Rhodobacterales	Rhodobacteraceae	Thalassobius		**SED**
Bacteria	Bacteroidetes	Cytophagia	Cytophagales	Flammeovirgaceae	uncultured 1059		**SED**
Bacteria	Proteobacteria	Deltaproteobacteria	Desulfobacterales	Desulfobulbaceae	Desulfotalea		**SED**
Bacteria	Proteobacteria	Deltaproteobacteria	Myxococcales	Sandaracinaceae	uncultured 11491		**SED**
Eukaryota	Ochrophyta	Diatomea	Coscinodiscophytina	Fragilariales	Licmophora		**SED**
Eukaryota	Ochrophyta	Diatomea	Bacillariophytina	Bacillariophyceae	Nitzschia		**SED**
Eukaryota	Dinoflagellata	Dinophyceae	Gymnodiniphycidae	Gymnodinium clade	Spiniferodinium		**SED**
Bacteria	Bacteroidetes	Flavobacteriia	Flavobacteriales	Flavobacteriaceae	Aquibacter		**SED**
Bacteria	Bacteroidetes	Flavobacteriia	Flavobacteriales	Flavobacteriaceae	Lutibacter		**SED**
Bacteria	Bacteroidetes	Flavobacteriia	Flavobacteriales	Flavobacteriaceae	Maribacter		**SED**
Bacteria	Bacteroidetes	Flavobacteriia	Flavobacteriales	Flavobacteriaceae	Muriicola		**SED**
Bacteria	Bacteroidetes	Flavobacteriia	Flavobacteriales	Flavobacteriaceae	Robiginitalea		**SED**
Bacteria	Bacteroidetes	Flavobacteriia	Flavobacteriales	Flavobacteriaceae	Ulvibacter		**SED**
Bacteria	Bacteroidetes	Flavobacteriia	Flavobacteriales	Flavobacteriaceae	Zeaxanthinibacter		**SED**
Bacteria	Proteobacteria	Gammaproteobacteria	BD7 8 marine group	Unknown 1002603	Unknown 1000892		**SED**
Bacteria	Proteobacteria	Gammaproteobacteria	Chromatiales	Ectothiorhodospiraceae	Unknown 1000902		**SED**
Bacteria	Proteobacteria	Gammaproteobacteria	E01 9C 26 marine group	Unknown 1002611	Unknown 1000908		**SED**
Bacteria	Proteobacteria	Gammaproteobacteria	Xanthomonadales	JTB255 marine benthic group	Unknown 1000984		**SED**
Bacteria	Chlorobi	Ignavibacteria	Ignavibacteriales	Ignavibacteriaceae	Ignavibacterium		**SED**
Eukaryota	Arthropoda	Insecta	Orthoptera	Unknown 1003417	Unknown 1001292		**SED**
Eukaryota	Ciliophora	Intramacronucleata	Spirotrichea	Hypotrichia	Holosticha		**SED**
Bacteria	Bacteroidetes	Sphingobacteriia	Sphingobacteriales	Saprospiraceae	Phaeodactylibacter		**SED**
Bacteria	Bacteroidetes	Sphingobacteriia	Sphingobacteriales	Saprospiraceae	uncultured 1272		**SED**
Bacteria	Bacteroidetes	Sphingobacteriia	Sphingobacteriales	S15 21	Unknown 1000338		**SED**
Eukaryota	Unknown 1003810	Unknown 1003811	Unknown 1003812	Unknown 1003813	Unknown 1001715		**SED**
Eukaryota	Ochrophyta	Diatomea	Coscinodiscophytina	Fragilariales	Licmophora	**SED**	**SH**
Eukaryota	Ochrophyta	Diatomea	Bacillariophytina	Bacillariophyceae	Nitzschia	**SED**	**SH, MJ**
Eukaryota	Unknown 1002968	Ulvophyceae	Unknown 1003017	Unknown 1003018	Unknown 1001105	**SEA**	**SH, MJ**

Core taxa based on richness found across all samples, sediment (SED) or seawater (SEA) environment types, and Sheltered (SH), Minor Outlet (MN), or Major Outlet (MJ) site locations. Core phyla and classes highlighted in yellow are specific to that environment type. Core genera highlighted in yellow indicate unique Operational Taxonomic Unit (OTU) numbers for repetitive names.

Bacterial Proteobacteria and eukaryal Florideophycidae displayed higher richness in seawater, whereas Bacterial Bacteroides and Planctomycetes and eukaryal Ciliophora and Annelida had higher richness in sediment; richness of all other archaeal-, bacterial-, and eukaryal phyla did not differ substantially between environment types (Fig 3, S2 Fig). Cytophagia (bacterial) and Chromadorea (eukaryal) were abundant classes specific to the sediment environment, whereas Cyanobacteria and Bacteroidia (bacterial) and Chlorophyceae (eukaryal) were specific to the seawater environment. A further breakdown of taxa richness by pairwise comparisons revealed that the microbial community taxa richness was the same across domains and locations for sediment (p > 0.1, f < 1.25). For seawater, archaeal and bacterial community composition (p = 0.017, f = 2.09 for 16S seawater subset; MJ-SH p = 0.084 / f = 2.45 and MJ-MN p = 0.021 / f = 2.80) was distinct for the MJ site and all sites were distinct for eukaryal community composition (p = 0.003, f = 2.28 for 18S seawater subset; MJ-SH p = 0.054 / f = 2.06, MJ-MN p = 0.031 / f = 2.69, SH-MN p = 0.025 / f = 2.02) (Fig 4, S3 Fig).

Investigation of the Puerto Nuevo ‘core’ taxa–those consistently found across environment types (sediment or seawater) and locations–resulted in 47 genera and 50 unique OTU identifications (Table 1, S7 and S8 Tables). For prokaryotic domains, only Bacteria were part of the Puerto Nuevo core taxa–no core Archaea were observed. Across the two domains (Bacteria and Eukarya), 13 phyla were observed. Actinobacteria, Cyanobacteria, and Phyla 1002968 were core phyla specific to the seawater environment, whereas Chlorobi, Arthropod, Ciliophora, and Phyla 1003810 were specific to the sediment environment. Proteobacteria, Orchophta, Dinoflagellata, and Bacteroides were core phyla shared between both environment types. Three bacterial classes and one eukaryal class were core to seawater, and four bacterial classes and three eukaryal classes were specific to sediment. Bacterial Alphaproteobacteria, Flavobacteriia, and Gammaproteobacteria and eukaryal Diatomea and Dinophyceae were shared core classes between sediment and seawater. Three genera were core taxa specific to the SH and MJ site locations, with two of the three genera specific to other categories (Puerto Nuevo core community taxa and sediment core community taxa). No core taxa were specific to the MN site location.

## Discussion

Microbial communities in the coastal Baja California region are understudied relative to Western coastal regions, and community dynamics among multiple domains within Baja California were unknown prior to this study. We characterized sample diversity within (alpha diversity) and between (beta diversity) coastal microbial communities by examining bacteria, archaea and microbial eukaryotes in both the sediment and seawater of Puerto Nuevo, Baja California. Our findings support the hypotheses that: (1) the variation in diversity is greater in coastal sediment microbial communities than seawater microbial communities along a 0.45 km range and (2) prokaryotic and eukaryotic microbial communities exhibit similar composition patterns in coastal sediment but different composition patterns in seawater. Our findings that coastal communities differ among sample sites and between environment type (sediment, seawater) are consistent with global patterns of microbial biodiversity; for example, studies on the Baltic Sea coastline and the coral reef systems of Indonesia find similar patterns as our study [60,61]. Furthermore, our observed differences for bacterial, archaeal, and eukaryal microorganisms between sites within a small 0.45 km range illustrate the necessity for future studies to expand geographical and temporal sampling in this region to better understand the microbial ecology and biodiversity patterns of Puerto Nuevo, Baja California.

The finding that the sediment environment type exhibits higher bacterial richness when compared to seawater is consistent with previous literature investigating bacterial diversity in and along the Pacific [45,62,63], with less being known in this regard for archaeal and eukaryal microorganisms. These results could be explained by the physical nature of the sediment environment type, allowing for an increase in the formation of microbial mats and biofilms by providing a surface for microorganisms to attach. In addition, the sediment is composed of minerals, and as such it supports the electric coupling of complex microbial redox reactions, which may serve important roles in biogeochemical cycling and the maintenance of ecological homeostasis [64]. In general, sediment is a stratified solid gradient that provides niche stability to microorganisms, whereas seawater is a dynamic liquid that is constantly in flux. These two environment types, however, are not mutually exclusive; the seawater environment type is a necessary contributor to refreshing the microbial populations within coastal environments [65], including the sediment. We note that we used different extraction kits for seawater and sediment. While we did include a blank as a control for the seawater extraction kit and observed little to no detectable DNA in the blank sample, there is always the possibility of different levels of bias from the sequencing results of the DNA samples extracted using different extraction methods. Nonetheless, we observed some overlap of core taxa between seawater and sediment across sites (Table 1), which suggests an interaction between these communities. Additionally, we observed consistency in microbial community composition between sites, which is particularly interesting for sediment samples, since sediment samples often display microspatial heterogeneity [66,67]. Overall, our study provides the framework for future studies to examine the microbial composition and taxa preferences between and among multiple environment types at a particular location site, and is a starting point for understanding the underlying functional implications that these preferences may play within specific ecosystems.

We observed distinct core taxa present for coastal Puerto Nuevo with three eukaryal genera specific to the sediment core taxa of one or more sampling sites (Table 1). Interestingly, *Nitzschia* and Unknown 1001105 were core genera (found in the sediment) that distinguish the Sheltered (SH) and Major Outlet (MJ) sites from the Minor Outlet (MN) site. *Nitzschia* has been found in regions with observed elevated nitrogen levels [68], and is a known toxin-producing diatom in marine and freshwater environments. *Licmophora* is another diatom which, unlike *Nitzschia*, is negatively impacted by human nitrogen pollutants [69] and could be in competition with *Nitzschia*. Interestingly, *Licmophora* is found in both the sediment and seawater whereas *Nitzschia* is only observed in the sediment (Table 1). Both *Nitzschia* and *Licmophora* were the only genera that showed up multiple times as distinguished core taxa for Puerto Nuevo microbial communities, be it sediment or seawater specific communities, or sampling site specific communities. Further investigation into the metabolomic profiles of these genera in relation to detailed biogeochemistry in the environments they are found may reveal novel information into the significance of these taxa in Puerto Nuevo and other coastal microbial communities.

We observed that different patterns of microbial taxa primarily depend on the environment type rather than the sampling site (Figs 3 and 4, Table 1). Akin to sediment hosting greater microbial biodiversity than seawater, we found a common pattern with previous literature in that beta diversity appears to be more important than alpha diversity in determining microbial community composition across environment types [70,71]. Many soil microbial ecology studies agree that drivers of microbial beta diversity vary across space. In specific reference to coastal and marine microbial communities, Barberán and Casamayor (2010) found that the significance of beta diversity and its drivers vary by phylum when specifically investigating bacterial Actinobacteria, Alphaproteobacteria, Bacteroidetes, Betaproteobacteria, Cyanobacteria, and Gammaproteobacteria [72]. This seems to be a common observation, affirmed by current studies in vastly different coastal microbial communities [73,74]. Puerto Nuevo sediment microbial communities within a 0.45 km range do not significantly differ between sites or domains (16S and 18S), whereas seaweater eukaryal microbial communities do demonstrate heterogeneity for all sites, and bacterial and archaeal microbial communities specifically differ for the Major Outlet site (Fig 4). Explanation for these results may be rooted in the physical dynamics of coastal seawater compared to sediment. Indeed, several studies demonstrate how the microbial community composition of aquatic and marine environments depend on scale [60,75,76], and while we did not explicitly test for scale, we observed statistically significant community composition variation to exist even for small 0.45 km ranges. Moreover, our study is consistent with previous studies observing the mixing of marine and terrestrial communities, where coasts are unique interfaces for comparing the two interacting environments. While more studies comparing coastal seawater and sediment are needed, especially for microbial eukaryotes, a recent study [77] on a coastal environment of Southern China found similar patterns as we have found in this study for Puerto Nuevo in that the environment type and geographic location impacted the community composition, a finding that is analogous to previous studies focused exclusively on bacterial communities [28,60,73,76,78,79]. Another recent study in China’s coastal waters reported on the biogeography of microbial eukaryotes [80], further adding to our knowledge of microbial community composition studies.

Overall, our study is consistent with other studies, while providing new information on microbial diversity for Puerto Nuevo. For example, studies in other locations [81,81,82] found that Chlorobi, a photosynthesizing bacterial phylum that is known to contribute to sulfur cycling, is generally present in the sediment. Our results also indicated that Chlorobi are present in the sediment of Puerto Nuevo. Also consistent with other studies in other locations, the photosynthesizing Cyanobacteria have been observed to exist preferentially in seawater [83–85], and we find this to be the case in Puerto Nuevo as well. In addition, Alphaproteobacteria and Gammaproteobacteria, which have been observed to be common phyla across multiple environment types in other regions [72,74,86,87], were also found in the sediment and seawater of Puerto Nuevo. While we do see archaea representative of abundant or rich taxa (Fig 3), we did not find any archaeal groups in the core taxa of Puerto Nuevo (Table 1). The lack of archaea in the core taxa of Puerto Nuevo is a novel finding in terms of marine microbial composition, and suggests that future studies should incorporate the inclusion of microbial eukaryotes in microbial community composition studies, as our results indicate that there is stronger co-occurrence between bacteria and microbial eukaryotes than between archaea and other domains.

## Conclusions

In this investigation, we have expanded our understanding of microbial diversity and community composition in a near-shore marine environment of Baja California–a coastal region that has been generally understudied. Our analysis of coastal microbial communities just North of Puerto Nuevo, Baja California, which combined 16S and 18S rRNA gene sequencing approaches of coastal seawater and sediment, identified strong relationships between sampling sites and environment types consistent with previous studies. Our findings also highlight the differences of small scale (0.45 km) beta diversity, and demonstrate the significance of integrating multi- domain, environment type, and sampling sites into microbial composition studies to provide ecological context to microbial biodiversity potentially impacted by human-induced climate change and development.

## Supporting information

S1 Table Taxa corresponding to 16S OTU data. Assigned prokaryotic taxonomy for 16S OTUs.
(CSV)

S2 Table Taxa corresponding to 18S OTU data. Assigned microbial eukaryotic taxonomy for 18S OTUs.
(CSV)

S3 Table Rarefied 16S reads for sediment and seawater samples. 16S reads, rarefied to 1000, for coastal Puerto Nuevo sediment and seawater samples.
(CSV)

S4 Table Rarefied 18S reads for sediment and seawater samples. 18S reads, rarefied to 1000, for coastal Puerto Nuevo sediment and seawater samples.
(CSV)

S5 Table Metadata for amplicon reads. Metadata and number of raw reads for 16S and 18S OTU sequences.
(CSV)

S6 Table Metadata for site locations. Metadata for coastal Puerto Nuevo site locations.
(CSV)

S7 Table Output table for 16S core taxa. Full output table for 16S core taxa found along coastal Puerto Nuevo.
(CSV)

S8 Table Output table for 18S core taxa. Full output table for 18S core taxa found along coastal Puerto Nuevo.
(CSV)

S1 Fig Metrics and rarefaction curves on read abundance. Read abundance histograms of prokaryotic 16S (a) and eukaryotic 18S (d) Simpson’s diversity, histograms of prokaryotic 16S (b) and eukaryotic 18S (e) Shannon’s diversity, and rarefaction curves of all prokaryotic 16S (c) and eukaryotic 18S (f) operational taxonomic units versus number of reads.
(TIFF)

S2 Fig Top 20 phyla per site location. Boxplots display top 20 (a) bacterial and archaeal 16S phyla and (b) eukaryal 18S phyla by richness.
(TIFF)

S3 Fig Microbial community variations per site location. Principal component analysis (PCA) plots of PC1 x PC2 show variation versus similarity of microbial communities between site locations for (a) bacterial and archaeal 16S in the sediment and (b) seawater and (c) eukaryal 18S in the sediment and (d) seawater.
(TIFF)
