# Supplementary figures and images for: Community ecology across bacteria, archaea and microbial eukaryotes in the sediment and seawater of coastal Puerto Nuevo, Baja California
Source: PLoS One. 2019 Feb 14;14(2):e0212355. doi: 10.1371/journal.pone.0212355 (PMC6375613; doi:10.1371/journal.pone.0212355)

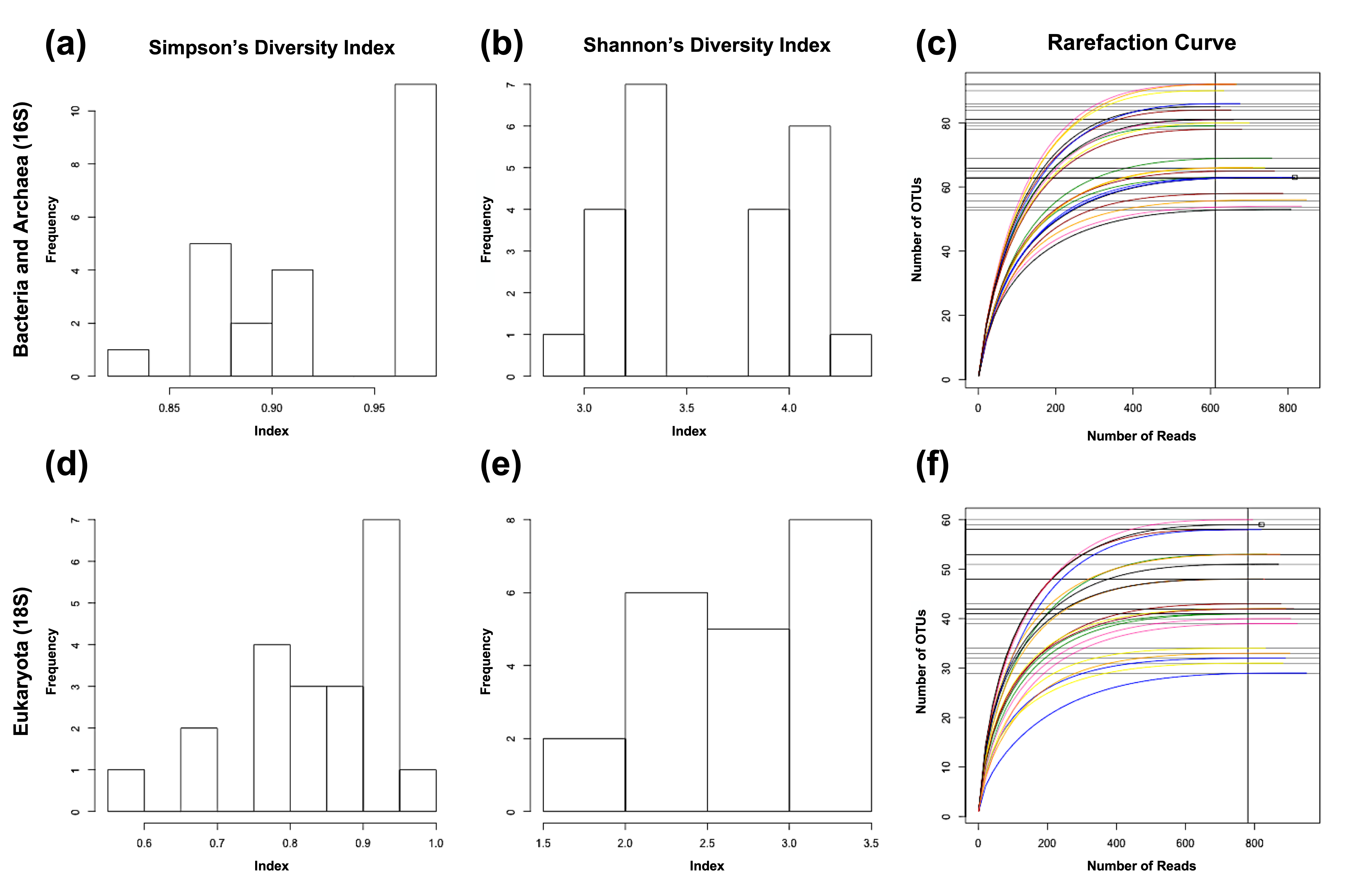

Supplement: S1 Fig — Read abundance histograms of prokaryotic 16S (a) and eukaryotic 18S (d) Simpson’s diversity, histograms of prokaryotic 16S (b) and eukaryotic 18S (e) Shannon’s diversity, and rarefaction curves of all prokaryotic 16S (c) and eukaryotic 18S (f) operational taxonomic units versus number of reads. (TIFF) [file pone.0212355.s009.tiff]

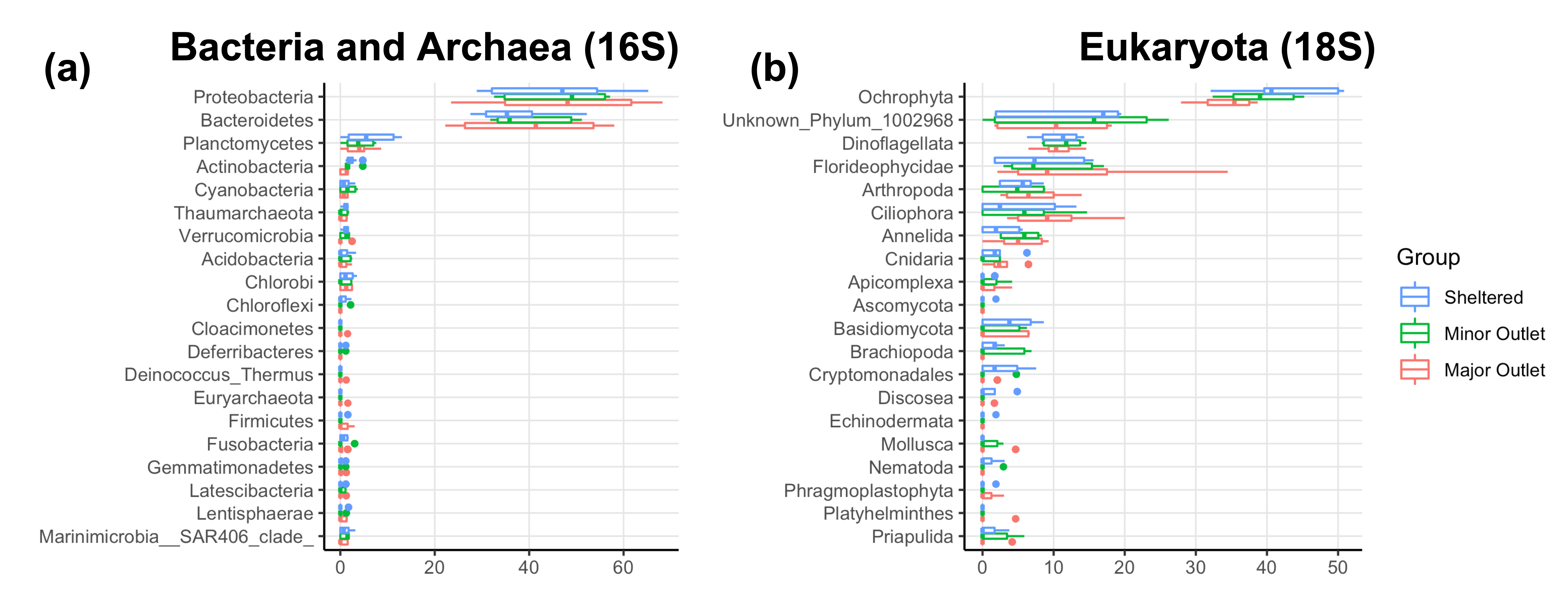

Supplement: S2 Fig — Boxplots display top 20 (a) bacterial and archaeal 16S phyla and (b) eukaryal 18S phyla by richness. (TIFF) [file pone.0212355.s010.tiff]

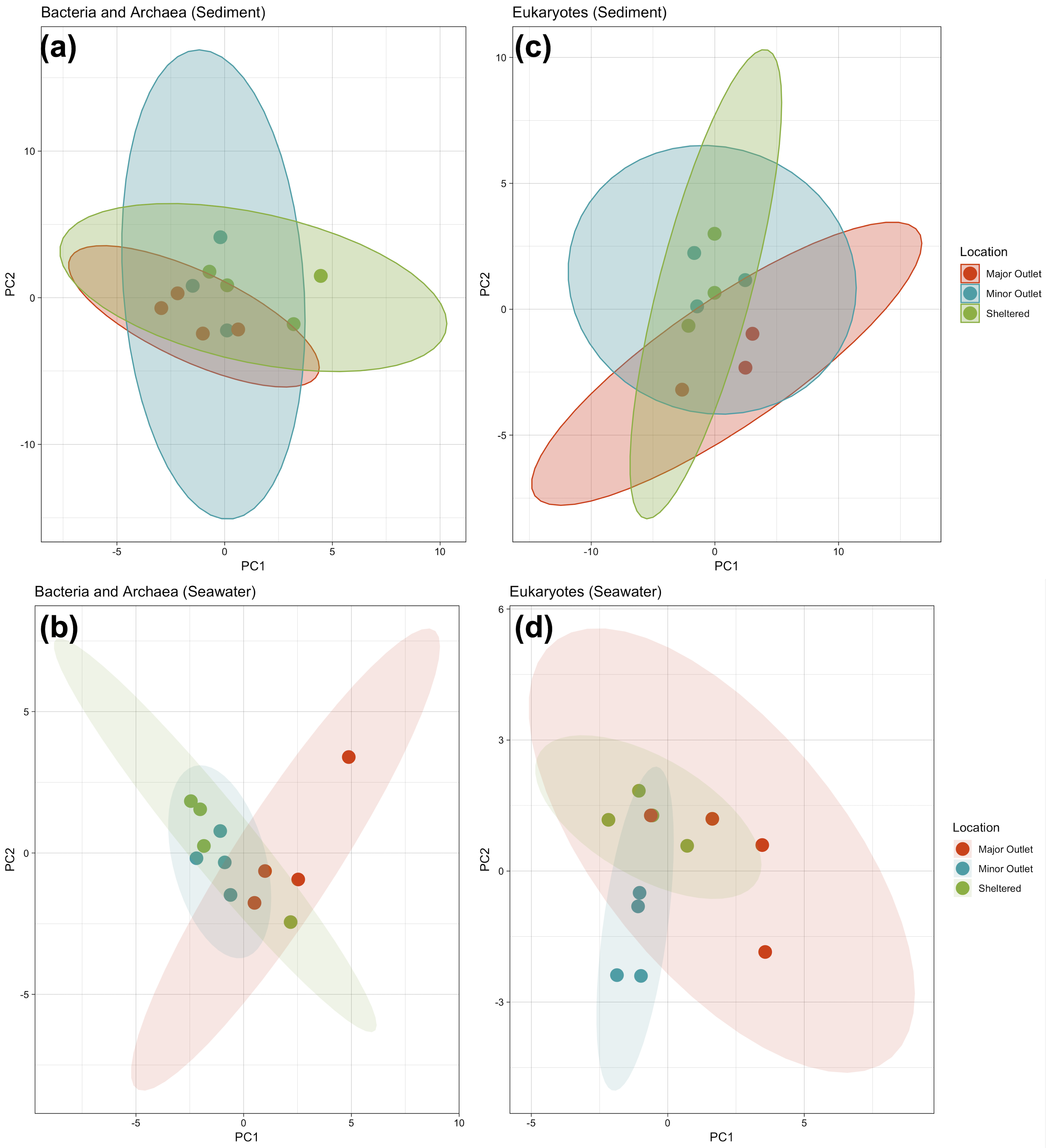

Supplement: S3 Fig — Principal component analysis (PCA) plots of PC1 x PC2 show variation versus similarity of microbial communities between site locations for (a) bacterial and archaeal 16S in the sediment and (b) seawater and (c) eukaryal 18S in the sediment and (d) seawater. (TIFF) [file pone.0212355.s011.tiff]
